# Learning to understand others' actions

**DOI:** 10.1098/rsbl.2010.0850

**Published:** 2010-11-17

**Authors:** Clare Press, Cecilia Heyes, James M. Kilner

**Affiliations:** 1 Wellcome Trust Centre for Neuroimaging, Institute of Neurology, University College London, 12 Queen Square, London WC1N 3BG, UK; 2 School of Psychology and Clinical Language Sciences, University of Reading, Whiteknights, Reading RG6 6AL, UK; 3 All Souls College and Department of Experimental Psychology, University of Oxford, High Street, Oxford OX1 4AL, UK

**Keywords:** mirror neuron, mirror system, associative sequence learning, predictive coding, action understanding

## Abstract

Despite nearly two decades of research on mirror neurons, there is still much debate about what they do. The most enduring hypothesis is that they enable ‘action understanding’. However, recent critical reviews have failed to find compelling evidence in favour of this view. Instead, these authors argue that mirror neurons are produced by associative learning and therefore that they cannot contribute to action understanding. The present opinion piece suggests that this argument is flawed. We argue that mirror neurons may both develop through associative learning *and* contribute to inferences about the actions of others.

## 1. Introduction

Mirror neurons, which have been discovered in the premotor area F5 [1] and inferior parietal lobule, area PF [2] of macaque monkeys, discharge not only when the monkey executes an action of a certain type (e.g. precision grip), but also when it observes the experimenter performing the same action. A number of neuroimaging studies have provided evidence that a similar system also exists in humans (e.g. [3]). A matter of much debate is whether activity in the so-called ‘mirror neuron system’ (MNS) reflects neural processes engaged in ‘action understanding’, that is, inferences about the goals and intentions driving an observed action. It has been suggested that mirror neurons are simply the result of learned sensorimotor associations, as proposed in the associative sequence learning (ASL) model [4,5], and that this ontogeny is inconsistent with a role in understanding the actions of others [6,7]. In contrast, we argue that mirror neurons may develop through associative learning *and* subsequently contribute to action understanding.

## 2. ASL model

**Figure 1.**
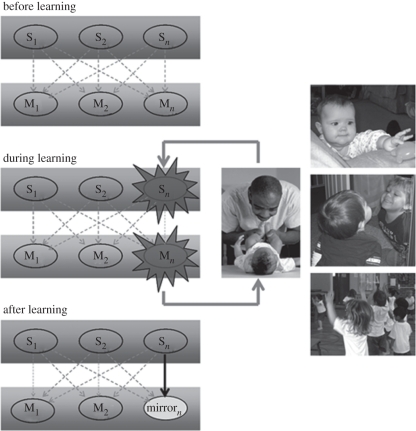
Associative sequence learning. Before learning, sensory neurons (S_1_, S_2_ and S_*n*_) which are responsive to different high-level visual properties of an observed action are weakly and unsystematically connected (dashed arrows) to some motor neurons (M_1_, M_2_ and M_*n*_), which discharge during the execution of actions. The kind of learning that produces mirror neurons occurs when there is correlated (i.e. contiguous and contingent) activation of sensory and motor neurons that are each responsive to similar actions.

The ASL model [4,5] proposes that the mirror properties of the MNS emerge through sensorimotor associative learning. Under this hypothesis, we are not born with an MNS. Rather, experience in which observation of an action is correlated with its execution establishes excitatory links between sensory and motor representations of the same action. We have abundant experience of matching relationships between observed and executed actions during our lives [8]. Following such experience, observation of an action is sufficient to activate its motor representation. Therefore, representations that were originally motor become ‘mirror’ (activated when observing and executing the same action, figure 1).

If the ASL model is correct, mirror neurons do not have an ‘adaptive function’, they did not evolve ‘for’ action understanding or to meet the demands of any other cognitive task [5]. However, as a by-product of associative learning, mirror neurons could still be recruited in the course of development to play some part in a variety of cognitive tasks. Therefore, according to the ASL model, they could be useful without being essential, and without their utility explaining their origins. Specifically, mirror neurons could play a part in action understanding even if this functional role was not favoured by natural selection in the course of phylogenetic evolution.

So why has the ASL hypothesis been interpreted as evidence against a functional role of mirror neurons in action understanding? Hickok [6] argued that some of the evidence that has been published in support of ASL is inconsistent with the hypothesis that the MNS is involved in action understanding. The studies in question require participants to observe actions while systematically executing non-matching actions, and subsequently record indices of MNS functioning. The rationale for these experiments assumes that, if the MNS develops through associative learning, then experiences that differ from those typically encountered during life should reconfigure the MNS and change the way it operates. Consistent with this prediction, it has been found that training in which participants are required to perform index finger actions when they see little finger actions, and vice versa, results in activation of primary motor cortical representations of the index finger when passively observing little finger actions, and activation of representations of the little finger when observing index finger actions [9,10]. Catmur *et al*. [11] demonstrated that such training effects are likely to be mediated by cortical circuits that overlap with areas of the MNS. They required one group of participants to lift their hand when they saw a hand lift, and to lift their foot when they saw a foot lift (matching group). Another group was required to lift their hand when they saw a foot lift, and to lift their foot when they saw a hand lift (non-matching group). Following such training, voxels in premotor and inferior parietal cortices that responded more when observing hand than foot actions in the matching group responded more to foot than hand actions in the non-matching group. This finding suggests that, following non-matching training, observation of hand actions activates motor representations of foot actions. Similar ‘counter-mirror’ training effects have also been observed in behavioural paradigms (e.g. [12,13], see also [14,15] for ‘logically related’ activations that may have been generated through naturally occurring non-matching experience).

Hickok [6] argued that these studies provide evidence that mirror neurons cannot underlie action understanding. Embracing the idea that counter-mirror training reconfigures the MNS—making it responsive to the sight of one action and the execution of a different action—he reasoned that, if the MNS contributes to action understanding, this reconfiguration should have an impact on action understanding. However, he considered that participants who showed counter-mirror activation (e.g. stronger activation of the index finger muscle during observation of little than of index finger movement) ‘presumably did not mistake the perception of index finger movement for little finger movement and vice versa’ ([6], p.1236). The key word here is ‘presumably’. Neither the focal study by Catmur *et al*. [9], nor any other study, has examined the effects of counter-mirror training on indices of action understanding.

## 3. Predictive coding and action understanding

The aim of the predictive coding (PC) account [16,17] was to answer the question ‘if mirror neurons enable the observer to infer the intention of an observed action, how might they do this’? In many accounts of the MNS, it is assumed that mirror neurons are driven by the sensory data and that when the mirror neurons discharge, the action is ‘understood’. However, within this scheme mirror neurons could only enable action understanding if there was a one-to-one mapping between the sensory stimulus and the intention of the action. This is not the case. If you see someone in the street raise their hand, they could be hailing a taxi or swatting a wasp. The context must establish which intention is more likely to drive an action. Consistent with the PC account, the empirical evidence does not support the view that mirror neurons are driven solely by sensory data from focal action stimuli. For example, Umilta *et al*. [18] found that neurons in F5, which fire both when the monkey executes and observes grasping actions, also fired when the monkey observed the experimenter's grasping action disappear behind a screen. That is, the premotor neurons represented a grasping action in its entirety, but where the grasping phase was not actually seen. Therefore, mirror neurons could not be driven entirely by the focal stimulus input. The PC account provides a framework that resolves these issues.

The essence of the PC account is that, when we observe someone else executing an action, we use our own motor system to generate a model of how we would perform that action to understand it [19,20]. PC enables inference of the intentions of an observed action by assuming that the actions are represented at several different levels [21] and that these levels are organized hierarchically such that the description of one level will act as a prior constraint on sub-ordinate levels. These levels include: (i) the intention level that defines the long-term desired outcome of an action, (ii) the goal level that describes intermediate outcomes that are necessary to achieve the long-term intention, (iii) the kinematic level that describes, for example, the shape of the hand and the movement of the arm in space and time. Therefore, to understand the intentions or goals of an observed action, the observer must be able to represent the observed movement at either the goal level or the intention level, having access only to a visual representation of the kinematic level.

**Figure 2.**
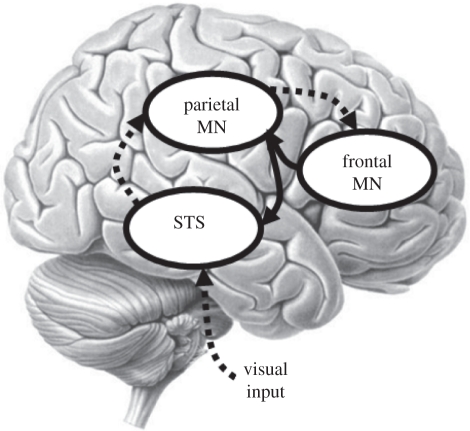
Predictive coding. Each level of the hierarchy predicts representations in the level below, via backward connections. These predictions are compared with the representations at the sub-ordinate level to produce a prediction error. This prediction error is then sent back to the higher level, via forward connections, to update the representation. By minimizing the prediction error at all the levels of the MNS, the most likely cause of the action will be inferred. Dotted line, prediction error; thick line, prediction.

PC proposes that contextual cues generate a prior expectation about the intention of the person we are observing. In the above example of the hand-raising action, these cues could be the presence of a taxi or wasp, or a facial expression. On the basis of these intentions, we can generate a prior expectation of the person's intermediate goals. Given their intermediate goals, we can predict the perceptual kinematics. Backward connections convey the prediction to the lower level where it is compared with the representation at this sub-ordinate level to produce a prediction error. This prediction error is then sent back to the higher level, via forward connections, to update the representation at this level (figure 2). By minimizing the prediction error at all the levels of action representation, the most likely cause of the action, at both the intention and the intermediate goal level, will be inferred. Thus, the PC process uses information, supplied by the MNS, about which goals are most likely, given a certain intention, and which kinematics are most likely, given a certain goal, to test hypotheses about the observed actors' intentions.

The assumptions of the PC model are consistent with those of ASL. If both models are correct, the MNS develops through associative learning and subsequently supports inferences about the goals and intentions driving others' actions. Therefore, it remains an open and important empirical question whether any intervention that systematically changes the MNS has correlated effects on action understanding.

## 4. Conclusion

PC and ASL accounts of the MNS address different questions and offer compatible answers. The PC account considers the requirements that are necessary to enable goal or intention inference during action observation. It assumes that the sensorimotor connection strengths have been learned, but does not propose a mechanism by which these are learned. ASL provides an associative mechanism for such learning. Although ASL does not provide a mechanistic account of how such learning could enable action understanding, it allows for the possibility that the MNS, once acquired, could support such functions. In other words, the MNS could enable inferences about the intentions of others, even if this function is not an evolutionary adaptation. Therefore, if both the PC and ASL hypotheses are correct, we learn, via the principles specified in associative learning theory, to predict others' intentions using our own motor systems.

## References

[1] Gallese V. Fadiga L. Fogassi L. Rizzolatti G. 1996 Action recognition in the premotor cortex. Brain 119, 593–609 8800951 10.1093/brain/119.2.593

[2] Fogassi L. Ferrari P. F. Gesierich B. Rozzi S. Chersi F. Rizzolatti G. 2005 Parietal lobe: from action organization to intention understanding. Science 308, 662–667 10.1126/science.1106138 (doi:10.1126/science.1106138) 15860620

[3] Kilner J. Neal A. Weiskopf N. Friston K. J. Frith C. 2009 Evidence of mirror neurons in human inferior frontal gyrus. J. Neurosci. 29, 10 153–10 159 10.1523/JNEUROSCI.2668-09.2009 (doi:10.1523/JNEUROSCI.2668-09.2009) PMC2788150 19675249

[4] Heyes C. 2001 Causes and consequences of imitation. Trends Cogn. Sci. 5, 253–261 10.1016/S1364-6613(00)01661-2 (doi:10.1016/S1364-6613(00)01661-2) 11390296

[5] Heyes C. 2010 Where do mirror neurons come from? Neurosci. Biobehav. Rev. 34, 575–583 10.1016/j.neubiorev.2009.11.007 (doi:10.1016/j.neubiorev.2009.11.007) 19914284

[6] Hickok G. 2009 Eight problems for the mirror neuron theory of action understanding in monkeys and humans. J. Cogn. Neurosci. 21, 1229–1243 10.1162/jocn.2009.21189 (doi:10.1162/jocn.2009.21189) 19199415 PMC2773693

[7] Hickok G. Hauser M. 2010 (Mis)understanding mirror neurons. Curr. Biol. 20, R593–R594 20656198 10.1016/j.cub.2010.05.047 PMC2911438

[8] Ray E. Heyes C. In press Imitation in infancy: the wealth of the stimulus. Dev. Sci. (doi:10.1111/j.1467-7687.2010.00961.x) 10.1111/j.1467-7687.2010.00961.x 21159091

[9] Catmur C. Walsh V. Heyes C. 2007 Sensorimotor learning configures the human mirror system. Curr. Biol. 17, 1527–1531 10.1016/j.cub.2007.08.006 (doi:10.1016/j.cub.2007.08.006) 17716898

[10] Catmur C. Mars R. B. Rushworth M. F. Heyes C. In press Making mirrors: premotor cortex stimulation enhances mirror and counter-mirror motor facilitation. J. Cogn. Neurosci. (doi:10.1162/jocn.2010.21590) 10.1162/jocn.2010.21590 20946056

[11] Catmur C. Gillmeister H. Bird G. Liepelt R. Brass M. Heyes C. 2008 Through the looking glass: counter-mirror activation following incompatible sensorimotor learning. Eur. J. Neurosci. 28, 1208–1215 10.1111/j.1460-9568.2008.06419.x (doi:10.1111/j.1460-9568.2008.06419.x) 18783371

[12] Heyes C. M. Bird G. Johnson H. Haggard P. 2005 Experience modulates automatic imitation. Cogn. Brain Res. 22, 233–240 10.1016/j.cogbrainres.2004.09.009 (doi:10.1016/j.cogbrainres.2004.09.009) 15653296

[13] Cook R. Press C. Dickinson A. Heyes C. 2010 The acquisition of automatic imitation is sensitive to sensorimotor contingency. J. Exp. Psychol. Hum. Percept. Perform. 36, 840–852 10.1037/a0019256 (doi:10.1037/a0019256) 20695703

[14] di Pellegrino G. Fadiga L. Fogassi L. Gallese V. Rizzolatti G. 1992 Understanding motor events: a neurophysiological study. Exp. Brain Res. 91, 176–180 1301372 10.1007/BF00230027

[15] Newman-Norlund R. D. van Schie H. T. van Zuijlen A. M. Bekkering H. 2007 The mirror neuron system is more active during complementary compared with imitative action. Nat. Neurosci. 10, 817–818 10.1038/nn1911 (doi:10.1038/nn1911) 17529986

[16] Kilner J. Friston K. J. Frith C. D. 2007 Predictive coding: an account of the mirror neuron system. Cogn. Process. 8, 159–166 10.1007/s10339-007-0170-2 (doi:10.1007/s10339-007-0170-2) 17429704 PMC2649419

[17] Kilner J. Friston K. J. Frith C. D. 2007 The mirror-neuron system: a Bayesian perspective. Neuroreport 18, 619–623 17413668 10.1097/WNR.0b013e3281139ed0

[18] Umilta M. A. Kohler E. Gallese V. Fogassi L. Fadiga L. Keysers C. Rizzolatti G. 2001 I know what you are doing: a neurophysiological study. Neuron 31, 155–165 10.1016/S0896-6273(01)00337-3 (doi:10.1016/S0896-6273(01)00337-3) 11498058

[19] Pobric G. Hamilton A. F. 2006 Action understanding requires the left inferior frontal cortex. Curr. Biol. 16, 524–529 10.1016/j.cub.2006.01.033 (doi:10.1016/j.cub.2006.01.033) 16527749

[20] Urgesi C. Candidi M. Ionta S. Aglioti S. M. 2007 Representation of body identity and body actions in extrastriate body area and ventral premotor cortex. Nat. Neurosci. 10, 30–31 10.1038/nn1815 (doi:10.1038/nn1815) 17159990

[21] Hamilton A. F. Grafton S. T. 2007 The motor hierarchy: from kinematics to goals and intentions. In The sensorimotor foundations of higher cognition (eds Haggard P. Rossetti Y. Kawato M.), Oxford, UK: Oxford University Press

